# Functional imaging in the zebrafish retinotectal system using RGECO

**DOI:** 10.3389/fncir.2013.00034

**Published:** 2013-03-06

**Authors:** Alison S. Walker, Juan Burrone, Martin P. Meyer

**Affiliations:** MRC Centre for Developmental Neurobiology, King's College LondonLondon, UK

**Keywords:** calcium indicator, RGECO, SyRGECO, zebrafish, *in vivo* imaging, direction selectivity, orientation selectivity

## Abstract

Genetically encoded calcium indicators (GECIs) allow repeated, non-invasive measurements of neural activity in defined populations of neurons, but until recently GECIs based on single fluorescent proteins have been limited to the green region of the color spectrum. Recent efforts in protein engineering have expanded the color palette of GECIs. One of these new GECIs, the red RGECO, is spectrally separate from the traditional GFP-based sensors such as GCaMP, and therefore opens the way for simultaneous, multicolor imaging of neural activity. While RGECO has been shown to report spontaneous calcium fluctuations in neurons, the precise relationship of RGECO signal to evoked-neural activity is not known. Measurements of neural activity using RGECO *in vivo* have also not been reported. Using dissociated hippocampal neurons we performed a systematic analysis of two forms of RGECO- a cytosolic form and a presynaptically localized form generated by fusion of RGECO to the presynaptic protein, synaptophysin (SyRGECO). We find that RGECO and GCaMP3 are comparable in terms of dynamic range, signal-to-noise ratios and kinetics but that RGECO is a more reliable reporter of single action potentials. In terms of performance SyGCaMP3 and SyRGECO are comparable, and both are more sensitive reporters of activity than the cytosolic form of each probe. Using the zebrafish retinotectal system we show that SyRGECO and RGECO are can report neural activity *in vivo* and that RGECO expression permits detailed structural analysis of neuronal arbors. We have exploited these attributes to provide a morphological and functional description of tectal cells selective for motion along the vertical axis. These results open up the possibility of using zebrafish to functionally image genetically defined pre- and postsynaptic circuit components, separable by color, which will be a powerful approach to studying neural interactions in the brain.

## Introduction

Genetically encoded calcium indicators (GECIs) permit repeated, non-invasive measurements of neural activity in defined populations of neurons *in vivo* (e.g., Mank et al., 2008; Tian et al., 2009; Lutcke et al., 2010; Nikolaou et al., 2012). They are therefore invaluable for studying long-term changes in neuronal activity associated with development, experience, and disease. The potential for GECIs to address long-standing questions in the field of neuroscience has certainly been a major driving force behind the development of GECIs with faster kinetics, increased sensitivity, dynamic range, and signal-to-noise ratios—attributes that are particularly desirable for studying neuronal function. Protein engineering of the GCaMP family of GECIs for example, has resulted in incremental improvements in some of these attributes so that a recent generation GCaMP, GCaMP3, is now widely used to study neural activity in a number of different model systems (Tian et al., 2009; Huber et al., 2012; Nikolaou et al., 2012). Despite these improvements in probe performance GECIs based on single fluorescent proteins have, until recently, been limited to the green region of the color spectrum. The color palette of available single wavelength GECIs has recently been expanded, however, and now includes a red-shifted indicator (R-GECO1; hereafter simply referred to as RGECO) based on a circularly-permuted mApple fluorophore (Zhao et al., 2011b). RGECO displays very little spectral overlap with GFP-based indicators such as the GCaMPs and this offers the potential for simultaneous, multicolor imaging of neural activity. While it has been demonstrated that RGECO is capable of reporting spontaneous calcium oscillations and large calcium transients triggered by chemically-induced depolarization, the precise relationship between RGECO responses and the number of action potentials (APs) is not known (Zhao et al., 2011b). As a result, thorough comparison of RGECO with existing probes is difficult. Furthermore, the use of RGECO to report neural activity *in vivo* has not been demonstrated. Here, we characterized RGECO response properties in neurons *in vitro* and *in vivo*, and further describe a presynaptically targeted form called synaptophysin-RGECO (SyRGECO). *In vitro* experiments performed in dissociated hippocampal neurons directly compared the response properties of both these probes with the existing green indicator GCaMP3 and its presynaptic targeted version, SyGCaMP3. We find that RGECO and GCaMP3 are comparable in terms of dynamic range, signal-to-noise ratios and kinetics but that RGECO is a more reliable reporter of single APs. We also provide evidence that the method of illumination can profoundly influence RGECO performance, and that this may underlie the discrepancy between our findings and those of a previous study (Yamada and Mikoshiba, 2012). We also show that the expression of RGECO and SyRGECO in the retinotectal system of the larval zebrafish can be used to report neuronal activation *in vivo*. Single tectal neurons expressing RGECO, or retinal ganglion cell (RGC) axons expressing SyRGECO responded to visual stimulation paradigms and allowed reliable and robust measurements of orientation- and direction-selectivity. These results suggest that RGECO is a viable reporter of neural activity that could be used in combination with established, GFP-based indicators such as the GCaMP family for two-color functional imaging *in vivo*.

## Materials and methods

### Generation of plasmid constructs

For *in vitro* studies, we used the cytomegalovirus (CMV) promoter to drive GECI expression. For *in vivo* studies, we made use of the Gal4:UAS system (Koster and Fraser, 2001). CMV:RGECO was obtained from Addgene (Addgene plasmid 32444). To generate CMV:SyRGECO and UAS:SyRGECO, RGECO was amplified with primers to introduce *SmaI* and *ClaI* sites and subcloned into the pCRBlunt II-TOPO shuttle vector (Invitrogen). The amplified RGECO gene was cut from the shuttle vector with *SmaI* and *ClaI* and directionally cloned into either the CMV:SyGCaMP3 or UAS:SyGCaMP3 vectors (Nikolaou et al., 2012), replacing GCaMP3 in each case. CMV:GCaMP3 was generated by PCR amplifying GCaMP3 with *BamHI* and *NotI* sites and directionally cloning the product into the *BamHI/NotI* sites of pEGFP-N2 (Clontech Laboratories) thus replacing the *EGFP* sequence. To generate the Huc:Gal4:UAS:RGECO plasmid the HuC promoter was excised from a HuC:GFP plasmid (gift of James Jontes, OHSU, USA) with *SacII* and *NotI* which was subsequently blunted by Klenow treatment. Gal4FF was excised from an ath5:Gal4 plasmid (Gift of Steve Wilson, UCL, UK) using *NotI* and *NcoI* which was also blunted with Klenow. The HuC and Gal4FF fragments were then triple ligated with a pEGFP-N2 plasmid (Clontech), which had been cut with *SacII* and *NotI*. The Huc-Gal4-Sv40 fragment was excised from the resulting plasmid using *SacII* and *AflII*, which was blunted with Klenow, and subcloned in pBS SK (+) which had been cut with *SacII* and *NotI* (blunted) to generate HuC-Gal4FF pBS SK. The UAS:RGECO fragment was subcloned as an *AflII-AseI* fragment (both ends blunted by klenow treatment) into the *EcoRV* site of HuC:Gal4FF pBS SK to generate HuC:Gal4:UAS:RGECO.

### Characterisation of RGECO and SyRGECO in dissociated hippocampal neuron cultures

Dissociated hippocampal cultures were prepared as described previously (Nikolaou et al., 2012). Plasmids coding for either GCaMP3, RGECO, SyGCaMP3, or SyRGECO, under the control of the CMV promoter, were co- or singly transfected at day 7 *in vitro* using Lipofectamine 2000 (Invitrogen). All experiments were performed on >14 days *in vitro* neurons. For extracellular field stimulation coverslips, on which neurons were cultured, were mounted in a custom-made chamber fitted with a pair of parallel platinum electrodes, 5 mm apart. During imaging and stimulation, neurons were incubated in HEPES-buffered saline (HBS; 139 mM NaCl, 2.5 mM KCl, 10 mM HEPES, 10 mM D-glucose, 2 mM CaCl_2_ and 1.3 mM MgCl_2_; pH 7.3 and 290 mOsmol) containing 0.025 mM amino-5-phosphonovaleric acid (APV) and 0.02 mM 6-cyano-7-nitroquinoxaline-2,3-dione (CNQX) at room temperature. Neurons were stimulated by delivering 1–2 ms, 80 V voltage pulses at 20 Hz, where each pulse approximates a single action potential (AP) (Zhao et al., 2011a). Single, 2, 5, 10, 20, 40, and 60 pulse stimulations were delivered, with multiple 10 AP stimulations interleaved during the time course of each experiment. One to twenty AP stimuli were pseudo-randomized, however, 40 and 60 AP stimuli were always delivered at the end of the stimulus sets to prevent possible activity-induced plasticity or rundown of responses. RGECO and GCaMP3 fluorescence signals from co-transfected neurons were recorded sequentially to avoid spectral cross–talk. Confocal imaging of hippocampal neurons was performed using an Olympus FV1000 confocal microscope equipped with a 40×/0.8 NA water-immersion objective (Olympus). Functional time-series were acquired at a rate of approximately 6 Hz and 0.2 × 0.2 μm resolution. RGECO was excited with a 543 nm laser line, with emission collected via a 560–660 nm band pass emission filter, whereas GCaMP3 was excited with a 488 nm laser line, with emission collected via a 505–525 nm band pass emission filter. Widefield images were obtained using an Olympus IX71 inverted microscope with a CCD camera (Coolsnap HQ) controlled by Slidebook software (Intelligent Imaging Innovations), equipped with a 40×/1.0 NA oil-immersion objective (Olympus). Functional time-series were acquired at a rate of approximately 6 Hz and with approximately 0.25 × 0.25 μm resolution. The excitation light source was a xenon-arc lamp (Lambda LS; Sutter Instruments), in which light exposure was regulated by a rapid shutter (smartShutter; Sutter Instruments) controlled by a Sutter Instruments lambda 10–3 controller, fitted with 470 ± 20 nm and 565 ± 22 nm band pass excitation, 515 ± 20 nm band pass and 590-nm long pass dichroic and 510 ± 15 nm band pass and 650 ± 36 nm band pass emission filters (Chroma Technology Corporation) for GCaMP3 and RGECO, respectively. Hippocampal data were analysed using custom written Matlab codes (Mathworks). Normalized signal intensity changes (ΔF/F) were calculated on time-series for each voxel. The maximum ΔF/F during the stimulus period for each voxel was used to generate summary images. For RGECO and GCaMP3, the mean of a square (10 × 10 voxels) region of interest (ROI) applied to summary images gave the peak response. As RGECO, but not GCaMP3, was expressed in the nucleus, ROIs were selected within the cytoplasmic region of the cell body. For SyGCaMP3 and SyRGECO-expressing neurons, ROIs were defined by an empirically derived threshold based on the summary images for the first 10 AP stimulation. Once defined these ROIs were applied to all other stimulations. In cases where rundown was observed, normalization was performed to generate stimulus-response curves based on the exponential regression of multiple 10 AP stimulations interspersed within each experiment. This enabled a correction factor to be applied to each stimulus timepoint. RGECO and GCaMP3 detection thresholds for single-APs were calculated as ΔF/F responses greater than three standard deviations of baseline noise. Rise and decay kinetics were calculated for 10 AP stimulations using semi-automated spike analysis software (Synaptosoft Inc.). In experiments where rundown occurred, kinetics were calculated from solely the first 10 AP stimulation to prevent bias from rundown. To determine the degree of co-localization of SyGCaMP3 and SyRGECO, puncta intensities of both probes were calculated within 7 × 7 voxel ROIs. To determine the degree of co-localization that arises through chance, analysis was also performed on datasets in which the SyGCaMP3 image was flipped vertically relative to the SyRGECO image.

### Microinjection and imaging of zebrafish larvae and neurite tracing

All *in vivo* experiments were performed in the pigmentation mutant, nacre which lacks all neural crest derived melanophores (Lister et al., 1999). On occasion, microinjections were performed on Tg(Isl2b:Gal4; UAS:SyGCaMP3) embryos. This transgenic line of zebrafish expresses SyGCaMP3 in RGCs. To generate transient and mosaic expression of RGECO in tectal cells a HuC:Gal4:5UAS:RGECO plasmid (50 ng/μl) in Danieau solution [1 M NaCl, 0.25 M HEPES, 30 mM Ca(NO_3_)_2_, 20 mM MgSO_4_] was microinjected into embryos at the 1–4 cell stage. This single plasmid contained the pan-neuronal promoter, HuC driving expression of the yeast transcriptional activator protein Gal4 directly upstream of the Gal4 DNA binding motif, the Upstream Activation Sequence (UAS) in frame with RGECO. In order to generate mosaic expression of SyRGECO in RGCs an activator plasmid containing Gal4 driven by an upstream HuC promoter (HuC:Gal4) was co-injected with an effector plasmid, where SyRGECO expression is driven by a UAS motif in frame with SyRGECO (UAS:SyRGECO). The effector and activator plasmids were both injected at a concentration of 25 ng/μl in Danieau solution. Plasmid DNA was prepared using miniprep kits (Qiagen). Zebrafish were maintained at 28.5°C on a 14 h ON/10 h OFF light cycle. Confocal imaging of visually-evoked RGECO and SyRGECO responses was performed using an LSM 710 confocal microscope equipped with a spectral detection scan head and a 20×/1.0 NA water-immersion objective (Carl Zeiss). Excitation was provided by a 543 nm laser line. Functional time-series were acquired at a rate of 6.5 Hz and 0.208 × 0.208 μm voxel resolution (256 × 256 voxels) and <2.1 AU pinhole aperture. Optical sections were obtained at <1.6 μm intervals and maximum intensity projections of RGECO and SyRGECO-expressing neurons were generated using NIH ImageJ. The Simple Neurite Tracer in FIJI (a processing package based on ImageJ released under the General Public License) was used to perform semi-automated tracing of z-stacks for 3D reconstruction of arbors and calculation of total branch lengths.

### Visual stimulation and analysis of orientation- and direction-selectivity

Visual stimulation and confocal imaging of zebrafish larvae *in vivo* were as described previously (Nikolaou et al., 2012). Briefly, larvae with mosaic expression of RGECO in tectal cells, and SyRGECO in retinal ganglion cells (RGCs) were restrained in 2% low melting point agarose, mounted dorsally onto a customized glass platform. The agarose was sufficient to restrain the larvae so that anesthesia was not required. Agarose was removed from in front of one eye, and the larva positioned with this eye facing a screen onto which visual stimuli were projected, while time-series were simultaneously captured from the contralateral tectum. The projected image filled a visual field of approximately 97° by 63°. Visual stimuli consisted of dark bars (8 cd/m^2^) (25% of mean) on a mean gray background (32 cd/m^2^). Each bar was 10° in width moving at 20°/s and separated from the preceding bar by 30°—enabling more than one bar on the screen at any one time. The long axis of the bar was orthogonal to the direction of motion. Bars were presented at 12 different directions evenly spaced across 360° and displayed in a pseudorandom order. A blank screen null condition of 2 s was also interleaved. Each inter-epoch interval was 8 s to enable the RGECO and SyRGECO signals to return baseline. Visual experiments were generated and controlled using custom written Labview and Matlab code (MathWorks) implemented on a ViSaGe stimulus presenter (Cambridge Research Systems, UK) and delivered via a DLP pico projector (Optoma). *In vivo* functional data were analysed as previously described (Nikolaou et al., 2012), excepting that median filtering was not performed in the image processing of this data.

### Whole-mount immunolabeling of larval zebrafish with single cell expression of RGECO and SyRGECO

Larval zebrafish were fixed with 2% paraformaldehyde (PFA) in phosphate-buffered saline (PBS) for 2 h at room temperature, and then treated with 0.25% Trypsin in PBS on ice for 20 min. The samples were blocked in 0.4% Blocking Reagent (Roche, Nutley, NJ) in PBS-T (1% Triton) for 2 h before incubating with primary antibodies (diluted 1:500 in 0.4% Blocking Reagent in PBS-T) for 2 days at 4°C. RGECO and SyRGECO were labeled by a rabbit polyclonal antibody which recognizes DsRed (Clonetech-632496). On experiments performed in the Tg(Isl2b:Gal4; UAS:SyGCaMP3) zebrafish larvae, SyGCaMP3 expression was amplified using a chicken polyclonal antibody which recognizes GFP (abcam-13970). Larvae were incubated for 1 day at 4°C in secondary antibodies Alexa Fluor 488 goat anti-chicken, 546 and anti-rabbit, and the nuclear stain TO-PRO-3 (Invitrogen-T3605) all diluted 1:500 in 0.4% Blocking Reagent in PBST. Labeled larvae were imaged as described above.

### Animals

All work in this study was approved by the local Animal Care and Use Committee (King's College London), and was carried out in accordance with the Animals (Experimental Procedures) Act, 1986, under license from the United Kingdom Home Office.

## Results

### *In vitro* characterisation of RGECO

**Figure 1:**
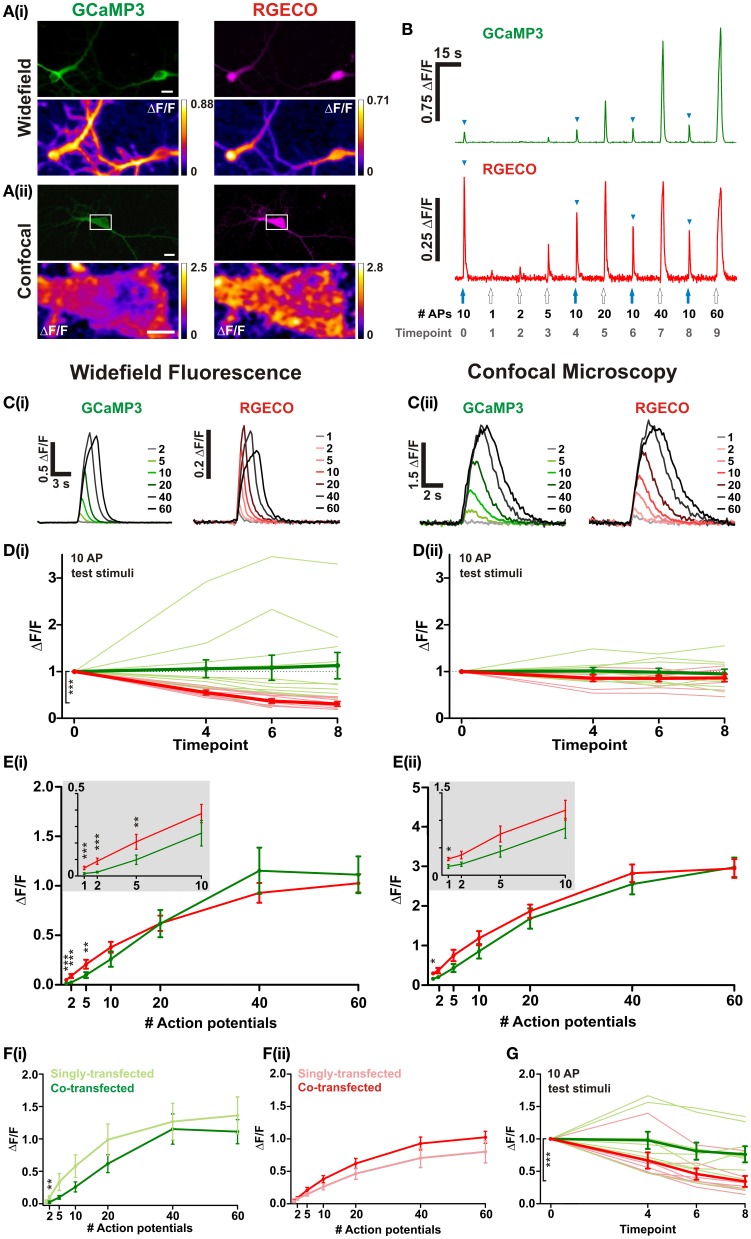
**Characterisation of RGECO and GCaMP3 expressed in hippocampal neurons.** For **(A–E)** neurons were co-transfected with RGECO and GCaMP3 to allow side-by-side characterization in the same neuron. **(A)** Images obtained using widefield **(i)** and confocal **(ii)** microscopy. Top panels show fluorescence images of GCaMP3 (green) and RGECO (magenta) co-transfected neurons; bottom panels show peak ΔF/F response summary images to 10 APs. Voxels are color-coded according to the maximum recorded ΔF/F (scale to the right). White boxes in top panel of **(ii)** indicate the area used for functional imaging in the bottom panel. Top panels scale bars = 10 μm; For **(ii)** bottom panel scale bar =5 μm. **(B)** Responses of RGECO and GCaMP3 from the same cell using widefield fluorescence to a typical experimental stimulation paradigm. The number of APs is indicated and blue arrowheads mark responses to interspersed repeats of 10 APs. **(C–E)** Characterisation of GCaMP3 and RGECO using widefield fluorescence **(i)** and confocal microscopy **(ii)**. **(C)** Mean responses of GCaMP3 and RGECO to a range of APs delivered at 20 Hz (widefield *n* = 8 cells; confocal *n* = 7). **(D)** Peak ΔF/F responses of RGECO and GCaMP3 to interspersed 10 AP test stimuli over the time course of the experiment (widefield *n* = 12 cells; confocal *n* = 7 cells). Mean responses and responses of individual cells are shown in bold and faint lines, respectively. For **(i)** note the significant rundown of RGECO responses (red traces) using widefield fluorescence, which is not seen with GCaMP3 (green traces), nor when using confocal microscopy **(ii)**. Kruskal-Wallis one-way ANOVA followed by Dunn's post-test. **(E)** Peak ΔF/F over AP number for RGECO and GCaMP3 (widefield *n* = 12 cells; confocal *n* = 7 cells). RGECO responses measured using widefield imaging are corrected for rundown (see section “Materials and Methods”). Insets show magnified region (1–10 APs) of plots and demonstrate that RGECO is better at reporting low spiking activity than GCAMP3. Wilcoxon signed rank tests. **(F)** Peak ΔF/F over AP number for GCaMP3 **(i)** and RGECO **(ii)** measured in single- and co-transfected neurons using widefield microscopy (single-transfected: GCaMP3 *n* = 7, RGECO *n* = 6; co-transfected: *n* = 12). Responses of RGECO were corrected for rundown (see section “Materials and Methods”). Mann Whitney test. **(G)** Mean peak ΔF/F responses of neurons singly-transfected with either GCaMP3 or RGECO (bold green and red, respectively) to interspersed 10 AP test stimuli over the timecourse of the experiment (GCaMP3 *n* = 7 cells; RGECO *n* = 6 cells). Responses of individual cells are denoted by thin green (GCaMP3) and red (RGECO) lines, respectively. Kruskal-Wallis One-Way ANOVA followed by Dunn's post-test. **(B–G)** GCaMP3-green traces; RGECO-red traces. Significance: ^*^ < 0.05, ^**^ < 0.01, ^***^ < 0.0001.

In order to directly compare the performance of RGECO with GCaMP3 both probes were co-transfected into dissociated hippocampal neurons. mApple, the fluorescent protein on which RGECO is based, exhibits a ~270-fold increase in bleaching rate under arc lamp illumination compared to the scanned laser illumination used in confocal imaging (Shaner et al., 2008). To examine whether the form of illumination influences the performance of RGECO, characterization was performed using both widefield fluorescence and confocal imaging. Expression of GCaMP3 and RGECO permitted visualization of neuronal morphology and, as previously reported (Yamada and Mikoshiba, 2012), GCaMP3 is excluded from the nucleus while RGECO is not (Figures 1A,B, top panels). Normalized changes in fluorescence (ΔF/F) were measured in response to varying numbers of APs delivered via extracellular field stimulation (1–2 ms, 80 V, 20 Hz). Typical peak ΔF/F response images are illustrated in the bottom panels of Figures 1A(i,ii), and concatenated fluorescence traces obtained from somatic ROIs in response to a typical stimulus set are shown in Figure 1B. To examine whether probe performance was stable over time, responses to a 10 AP test stimulus were measured at regular intervals during each experiment. Under widefield illumination RGECO responses to these test stimuli showed an exponential decrease in amplitude, a trend not shown by GCaMP3 expressed in the same neuron [Figures 1B,D(i)]. This progressive decrease in RGECO signal, which we will refer to as rundown, is also clearly evident in the mean RGECO traces to 40 and 60 APs which are delivered toward the end of the stimulus set, and have lower peak values than responses recorded for fewer numbers of APs delivered earlier in the stimulus set [Figure 1C(i)]. This contrasts with GCaMP3 where the peaks of the mean response traces increase steadily with stimulus strength until they plateau at approximately 40 APs [Figures 1C(i),E(i)]. By applying a correction factor derived from the exponential rundown of RGECO to the RGECO responses (see section “Materials and Methods”) we were able to generate a stimulus-response curve for this probe that mirrored that of GCaMP3 in terms of dynamic range [Figure 1 E(i)]. When identical experiments were performed using confocal microscopy we did not observe any rundown in RGECO responses over time [Figure 1D(ii)]. As a result no correction of the data was required, and the responses of RGECO to varying numbers of APs display a dynamic range highly similar to that of GCaMP3 [Figures 1C(ii),E(ii)]. In addition, RGECO proved to be a more robust reporter of single APs than GCaMP3 [Figure 1E (insets)]. Responses to single spikes measured using confocal microscopy (which does not suffer from potential correction-based errors) showed that RGECO detects 1 AP in 88% of trials, compared to only 38% for GCaMP3 (see section “Materials and Methods”). Furthermore, the kinetics of RGECO and GCaMP3 to 10 AP stimuli were very similar (½t rise: GCaMP3, 566 ± 39 ms and RGECO, 511 ± 26 ms; ½t decay: GCaMP3, 1020 ± 51 ms and RGECO, 1039 ± 60 ms). Lastly, we performed these experiments on neurons transfected with either RGECO or GCaMP3 alone. In singly transfected neurons the response magnitudes, sensitivity, and dynamic range of GCaMP3 and RGECO were similar to those measured in co-transfected neurons [Figures 1F(i,ii)]. As with co-transfected neurons, RGECO exhibited rundown in singly transfected cells imaged with widefield microscopy (Figure 1G).

These results suggest that the prolonged and continuous excitation of RGECO under widefield fluorescence imaging severely compromises its ability to faithfully report levels of neuronal activity. However, when using confocal imaging and scanned excitation RGECO does not show the rundown seen with widefield imaging, and performs as well as GCaMP3 at reporting stronger stimuli while demonstrating greater sensitivity to single APs.

### *In vitro* characterisation of SyRGECO

**Figure 2:**
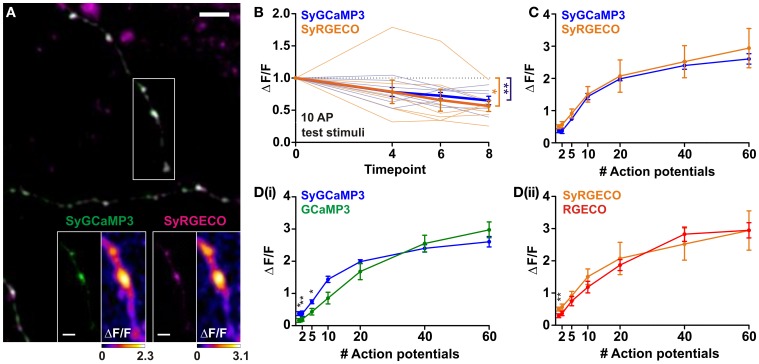
**Characterisation of SyGCaMP3 and SyRGECO in hippocampal neurons. (A)** Dissociated hippocampal neurons co-expressing SyGCaMP3 (green) and SyRGECO (magenta). Scale bar = 5 μm. White box indicates area used for functional imaging in SyGCaMP3 and SyRGECO insets. Right hand panels of insets show peak ΔF/F response summary images to 10 APs. Voxels are color-coded according to the maximum recorded ΔF/F (scales below). Scale bars for insets = 2 μm. **(B)** Peak ΔF/F responses for SyGCaMP3 and SyRGECO to interspersed 10 APs test stimuli throughout the experiment. Mean responses and individual cell responses are shown in bold and faint lines, respectively (*n* = 7 cells). Kruskall-Wallis One-Way ANOVA followed by Dunn's post-test. **(C)** Peak ΔF/F over AP number for SyGCaMP3 and SyRGECO (*n* = 7 cells). Responses are corrected for rundown (see section “Materials and Methods”). **(D)** Comparison of the peak ΔF/F over AP number for SyGCaMP3 and cytosolic GCaMP3 **(i)**; and for SyRGECO and RGECO **(ii)** using confocal microscopy (SyGCaMP3 *n* = 7; GCaMP3 *n* = 12, SyRGECO *n* = 7; RGECO *n* = 12). SyGCaMP3 and SyRGECO were both corrected for rundown (see section “Materials and Methods”), as cytosolic GCaMP3 and RGECO did not exhibit rundown using confocal microscopy (see Figure 1) they were not corrected. Mann whitney tests. Significance: ^*^ < 0.05, ^**^ < 0.01.

One of the strengths of using GECIs is the ability to target their expression to specific subcellular compartments. Here, we have used a previously described strategy (Dreosti et al., 2009) to restrict probe expression to presynaptic terminals through fusion of GCaMP3 and RGECO to synaptophysin, a synaptic vesicle protein. Targeting GECIs to synaptic boutons allows recording of calcium transients which specifically trigger neurotransmitter release. Synaptophysin-GCaMP3 (SyGCaMP3) has been previously characterized using widefield fluorescence microscopy (Nikolaou et al., 2012), while synaptophysin-RGECO (SyRGECO) is a newly generated probe. Using widefield fluorescence imaging we found that the rundown of SyRGECO responses was so rapid that we were unable to characterize the probe using this form of microscopy. Instead, SyGCaMP3 and SyRGECO were characterized side-by-side in co-transfected dissociated hippocampal neurons using confocal microscopy. In these neurons both probes exhibited a punctate pattern of expression (Figure 2A), with 97% of SyRGECO puncta co-localizing with SyGCaMP3 (157 puncta from 4 cells; data not shown), which has previously been shown to be localized to presynaptic terminals *in vitro* (Nikolaou et al., 2012). When stimulated, the peak response (ΔF/F) is mainly localized to presynaptic boutons with much lower signals produced in the adjoining axon (see insets in Figure 2A). Using the same stimulation paradigms used to characterize cytosolic RGECO we found that responses of both SyGCaMP3 and SyRGECO were not stable over time, with both exhibiting an approximately 40% rundown in response to 10 AP test stimulations interspersed throughout the experiment (Figure 2B). Application of a correction factor derived from the slope of the rundown led to the generation of stimulus-response curves. We found that SyGCaMP3 and SyRGECO exhibited very similar responses at all stimulus strengths both in terms of response magnitude (Figure 2C) and kinetics to 10 AP stimuli (½t rise: SyGCaMP3, 409 ± 50 ms and SyRGECO 401 ± 46 ms; ½t decay: SyGCaMP3, 901 ± 96 ms and SyRGECO, 951 ± 105). In comparison to the cytosolic forms, we find that SyGCaMP3 exhibits greater sensitivity to low numbers of APs (1–5 APs) compared to GCaMP3. While SyRGECO is more sensitive to single spikes than cytosolic RGECO, the stimulus-response curve of SyRGECO more closely resembles that of cytosolic RGECO (Figure 2D). These results demonstrate that RGECO, when targeted to presynaptic terminals, retains its high sensitivity and large dynamic range.

### Characterisation of single orientation-selective tectal cells *in vivo* using RGECO

**Figure 3:**
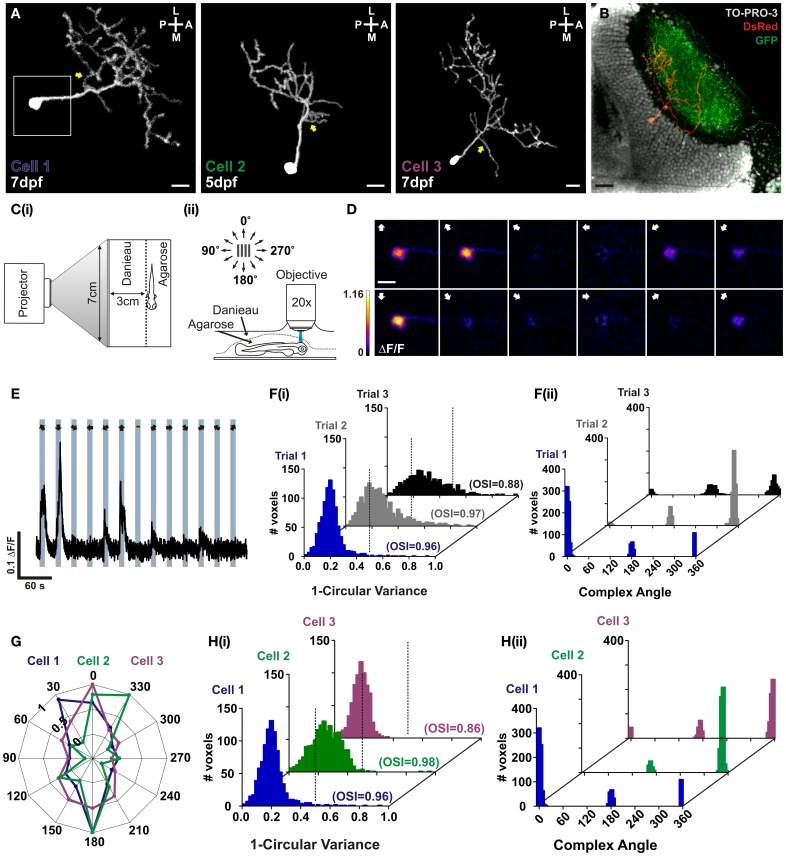
***In vivo* characterization of orientation-selective tectal neurons expressing RGECO. (A)** Dorsal view of three example volumetric fills (see section “Materials and Methods”) of tectal neurons expressing RGECO at either 5- or 7- days post fertilization (dpf), as labeled. Image orientation is shown top right (L, lateral; M, medial; A, anterior; P, posterior). Yellow arrows indicate short proximal branches emanating from primary dendrites. Scale bars = 10 μm. White box indicates the area imaged for **(D)**. **(B)** *Post-hoc* immunostaining of a Tg(Isl2b:gal4:UAS:SyGCaMP3) zebrafish in which retinal ganglion cells express SyGCaMP3, with RGECO expression in a single tectal cell (Cell 3). Scale bar = 20 μm. **(C)** Schematic detailing the experimental set-up. **(i)** Larvae are immobilized in agarose, with one eye viewing a projection screen. **(ii)** Visually evoked responses are recorded in the contralateral tectum. **(D)** Montage of summary integral ΔF/F response images to a bar drifting in 12 different directions. The direction of motion is indicated by arrows in the top left of each panel. Voxels are color-coded to the maximum recorded integral ΔF/F (scale to the left). Scale bar = 10 μm. **(E)** Representative ΔF/F trace of a single voxel during a tuning experiment for Cell 1. Stimulus epochs are shown in blue with the direction of motion indicated by arrows (dash indicates the “blank” epoch). **(F)** Quantative voxel-wise analysis of the orientation-selectivity of Cell 1 across repeated trials. **(i)** Distribution of 1-circular variance for responsive voxels for each trial, as labeled. Voxels with values less than 0.5 (dotted line) are considered orientation-selective. Mean orientation-selective index (OSI) values are shown bottom left of each example histogram. **(ii)** Distribution of the complex (preferred) angle of all voxels in **(i)**. **(G)** Representative color-coded polar plots of single voxel integral responses of example cells (Cells 1–3). **(H)** Quantative voxel-wise analysis of orientation-selectivity for example tectal cells. **(i)** Distribution of 1-circular variance for responsive voxels for example cells [color-coding as in **(E)**]. Mean OSI values are shown bottom left of each example histogram. **(ii)** Distribution of the complex (preferred) angles of all voxels in **(i)**.

In order to characterize RGECO *in vivo* we labeled single tectal neurons in the larval zebrafish using mosaic labeling techniques and imaged calcium signals in response to visual stimulation. As well as reporting visually evoked activity, RGECO expression also permitted analysis of tectal cell morphology (Figure 3A). Furthermore, RGECO fluorescence could be amplified by *post-hoc* immunostaining with a DsRed antibody to provide detailed structural analysis. This also allowed for co-labeling of tectal landmarks such as RGC axons and tectal cell nuclei so that cell body and dendritic arbor positions of single RGECO expressing neurons could be analysed (Figure 3B). For functional imaging, zebrafish larvae were restrained in agarose with one eye facing a screen onto which dark bars were projected that moved across the visual field in 12 different directions (Figure 3C). The long axis of the bar was orthogonal to the direction of motion. Tectal soma responses to visual stimulation were captured by confocal imaging of the contralateral tectum. Figure 3D shows a montage of a tuning experiment performed on a single neuron (Cell 1) in which each voxel is color-coded according to the integral response at each stimulus direction. Figure 3E shows a representative ΔF/F trace of a single voxel from Cell 1 during a tuning experiment. For the example shown, RGECO reported selective fluorescent increases for motion along the vertical axis (Figure 3D). This was measured explicitly for each responding voxel using two measures of orientation-tuning: 1-circular variance and the mean orientation-selective index (OSI), with the complex angle providing the preferred angle for orientation-selective responses. Both measures revealed Cell 1 to be highly selective (1-circular variance< 0.5, OSI> 0.5) for motion along the vertical axis (Figure 3F). To examine whether rundown of RGECO occurred during *in vivo* imaging we performed three consecutive tuning experiments. We reasoned that significant rundown of RGECO during the course of an experiment could cause trial-to-trial differences in tuning curves because the direction of motion is randomized for each trial, meaning that any rundown could artificially boost the response amplitude to the first presented direction relative to the last. However, we find that all three trials revealed cell 1 to be highly orientation-selective with an invariant complex angle showing selectivity for motion along the vertical axis (Figure 3F).

We found that our expression strategy often labels tectal cells that are orientation-selective for motion along the vertical axis (Figures 3G,H). We were interested in whether these functionally similar cell types also shared morphological traits. For all 3 cells studied here, we found a small proximal branch that extends from the primary dendrite (Figure 3A yellow arrows) and an asymmetrical elaboration of the dendritic tree in the posterolateral quadrant. These dendrites were found in the deeper portion of the stratum fibrosum et griseum superficiale (SFGS) of the tectal neuropil but were not strictly laminar in structure. In addition, the two age-matched examples, cell 1 and cell 3, showed comparatively large dendritic trees extending 95.23 μm and 91.81 μm respectively across the anterior–posterior axis of the tectum, and total branch lengths of 854 μm and 884 μm respectively.

These results demonstrate that RGECO can be used to report neuronal morphology and function *in vivo*. We have exploited these attributes to provide the first morphological and functional description of orientation-selective tectal cells in the zebrafish optic tectum.

### SyRGECO reports the direction-selectivity of a single RGC *in vivo*

**Figure 4:**
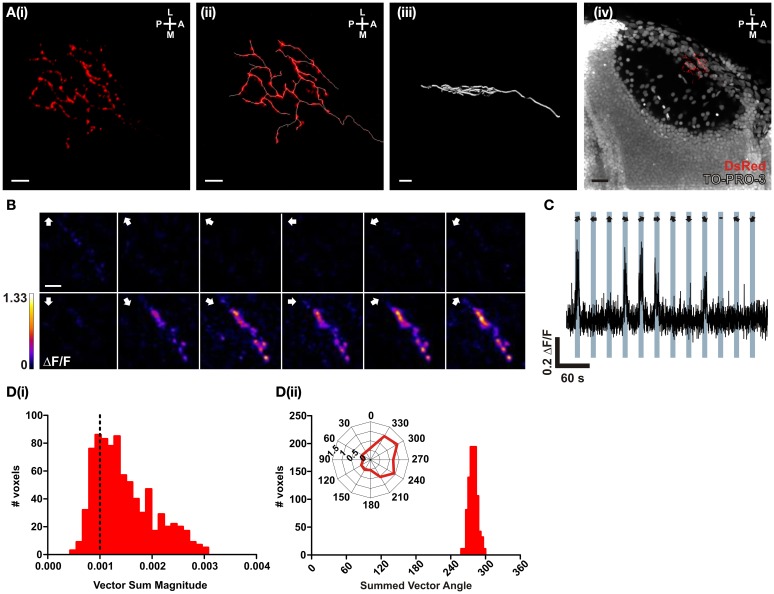
***In vivo* characterization of a direction-selective RGC expressing SyRGECO. (A)** A typical RGC axon expressing SyRGECO arborizing in the tectum at 6 dpf; **(i)** SyRGECO immunolabeled with an anti-DsRed antibody, **(ii)** immunolabeled RGECO overlaid with a trace for arbor morphology, **(iii)** side-on view of laminar arbor organization, **(iv)** nuclear staining with TOPRO-3 reveals relative RGC arbor position within the tectum. Image orientation is shown in top right for (L, lateral; M, medial; A, anterior; P, posterior). For **(i–iii)** scale bar = 5 μm; **(iv)** scale bar = 20 μm. **(B)** Montage showing typical integral ΔF/F responses of all voxels in the imaging region. Direction of motion is indicated by arrows in the top left of each panel. Voxels are color-coded to the maximum recorded integral ΔF/F (scale to the left). Scale bar = 3 μm. **(C)** Representative ΔF/F trace of a single voxel during a tuning experiment. Stimulus epochs are shown in blue with the direction of motion indicated by arrows (dash indicates the “blank” epoch). **(D)** Quantative voxel-wise analysis of direction-selectivity. **(i)** Distribution of vector sum for responsive voxels. Voxels larger than 0.001 (dotted line) are considered direction-selective. **(ii)** Distribution of the preferred angle of all suprathreshold direction-selective voxels in **(i)**. Representative polar plot shows the integral responses of a single voxel to the labeled directions of motion.

In order to test the performance of SyRGECO *in vivo*, calcium transients in the axon arbor of a singly-labeled RGC expressing SyRGECO were measured in response to visual stimulation. The labeled axon showed a punctate distribution of SyRGECO, consistent with a presynaptic localization and the axon arbor showed a classic planar morphology (Figure 4A). On presentation of a moving bar, stimulus-locked selective fluorescence increases were recorded (Figure 4C) which were confined to the regions of SyRGECO expression (Figure 4B). A montage of a tuning experiment in which each voxel is color-coded according to the integral response at each stimulus direction suggests selectivity for anterior (tail-to-head) motion (Figure 4B). This was examined closer by calculating the vector magnitude of each responding voxel with application of an empirically derived vector sum magnitude threshold (>0.001) to distinguish direction-selective from non-direction-selective voxels. For the cell shown, the majority of voxels are suprathreshold [Figure 4D(i)] with a mean summed vector angle of 280° [Figure 4D(ii)], which demonstrates selectivity for anterior motion. These results demonstrate that when fused to synaptophysin, RGECO (SyRGECO) can be used *in vivo* to report presynaptic activity in response to physiologically relevant stimuli.

## Discussion

The purpose of this study was to characterize the newly engineered red-shifted GECI RGECO in response to different numbers of APs *in vitro*, to generate a red-shifted synaptically-targeted GECI based on RGECO, and to determine whether RGECO is a useful reporter of neural activity *in vivo*, which has previously not been demonstrated.

In terms of performance *in vitro* we found that RGECO was comparable to the GFP-based GECI, GCaMP3, when imaged using confocal microscopy. Dynamic range, response magnitude and kinetics were not significantly different between RGECO and GCaMP3 when co-expressed in the same cells. Furthermore, co-expression did not alter the performance of either probe relative to cultures transfected with a single probe. This suggests that RGECO is not only useful for reporting a range of activity patterns but that it can also be used in combination with the more traditional GFP-based reporters of neural activity such as GCaMP family members (Tian et al., 2009; Muto et al., 2011; Akerboom et al., 2012) and synaptopHluorins (Miesenbock et al., 1998). Indeed, RGECO showed a greater sensitivity than GCaMP3 for detecting single spikes, an important advantage as this metric, in addition to linearity, is essential for the deconvolution of complex calcium signals into spikes (Yaksi and Friedrich, 2006; Dreosti et al., 2009). Furthermore, we found that RGECO, when fused to synaptophysin, acted as a good reporter of calcium influx at synaptic boutons with a similar dynamic range, magnitude of response and kinetics as SyGCaMP3.

Our findings contrast with those of a previous study in which RGECO was found to perform poorly in comparison to GCaMP3 (Yamada and Mikoshiba, 2012). During the course of our investigation we have discovered that differences in methods of RGECO excitation could provide a likely explanation for this discrepancy. We consistently observed light-dependent rundown in RGECO responses under widefield excitation, where under prolonged periods of widefield illumination RGECO becomes increasingly unreliable in reporting neural activity. Because these experiments were performed on neurons also expressing GCaMP3, which did not display rundown, we can rule out neuron ill-health or activity-induced plasticity as the cause of reduced RGECO responses. RGECO rundown was not observed when using the scanned illumination used in confocal microscopy, suggesting that either the method or degree of illumination can influence the response properties of RGECO. The confocal frame scan rate used in our study was fairly modest (6 Hz) and was targeted to the cell soma. Yamada and Mikoshiba (2012) characterized RGECO using rapid (200 Hz) 2-photon line-scanning over a region of apical dendrite. The RGECO excitation-emission cycling rates are therefore likely to be far higher in the Yamada study than in ours. In general, RGECO rundown is also likely to be exacerbated by imaging in low volume regions such as dendritic and axonal processes where there is less naïve probe available to replenish the light-depleted pool. This may underlie the rundown observed here of SyGCaMP3 and SyRGECO which are confined to the presynaptic terminal. Tethering these probes to synaptic vesicles will also limit probe motility and hence recovery from the rundown by probe turnover.

The basis for the light-induced rundown of RGECO responses is not clear. mApple, the fluorophore on which RGECO is based, bleaches far more quickly under widefield illumination than under confocal scanning (Shaner et al., 2008) indicating sensitivity to the method of illumination. However, photobleaching cannot fully explain RGECO rundown as we did not see significant bleaching of RGECO in our experiments and our response metric (ΔF/F) specifically normalizes for baseline fluorescence. This suggests that rundown may instead be due to a light-induced change in either the ability to bind calcium or to exhibit calcium-dependent increases in fluorescence. Whatever its underlying causes, the light-dependent rundown of RGECO means that this probe should be used with caution and with the appropriate controls.

To examine the performance of RGECO *in vivo* we used the retinotectal projection of zebrafish as a model system. While the broad function of the tectum is known: it converts a visuotopic map into motor commands that orient the head and body toward or away from a visual stimulus (for review see Nevin et al., 2010), the specific computations performed by local tectal circuits are not well understood. This is due to our relatively poor understanding of the essential circuit components: the various tectal cell types, their morphologies, functional properties and how cell types interact with one another (but see Robles et al., 2011; Gabriel et al., 2012 for examples). Expression of GECIs in single, identifiable cell types are well suited to addressing these gaps in our knowledge. In zebrafish it is also relatively straightforward to label and image single neurons and to provide visual stimuli to probe the functional properties of labeled cells (Dreosti et al., 2009; Gabriel et al., 2012; Nikolaou et al., 2012). Here we expressed SyRGECO in single RGCs and cytosolic RGECO in single tectal cells in larval zebrafish and used a drifting bar stimulus to functionally image two cell types: a direction-selective RGC with preference for tail-to-head motion, and orientation-selective tectal cells with a preference for motion along the vertical axis. In three successive tuning experiments performed on the same RGECO expressing tectal cell we saw that three measures of orientation tuning: 1-circular variance, orientation-selective index and complex angle were relatively invariant suggesting that RGECO rundown was not significantly altering the ability to measure tuning profiles *in vivo*. In order to classify neurons they must be defined using multiple criteria including morphology, molecular markers, patterns of connectivity and function. Our results suggest that RGECO and SyRGECO may be used to define two of these criteria; morphology and function, simultaneously. The direction-selective RGC that we describe here is located within the superficial tectal neuropil which matches the location of anterior-selective RGC axons described in a functional population study of RGC inputs to the tectum (Nikolaou et al., 2012). SyRGECO expression allowed for reconstruction of axonal arbor morphology, identification of putative presynaptic terminals and placement of the axon arbor within the tectal neuropil. The RGECO expressing tectal cells presented here demonstrate strikingly similar functional properties but also have morphological features in common. The somata of the orientation-selective tectal cells we have imaged are located quite superficially in stratum periventriculare with dendritic arbors that, although not strictly laminar, target the deeper layers of SFGS—a position that matches the location of vertically tuned orientation-selective RGC inputs into the tectum (Nikolaou et al., 2012). This suggests that excitatory, vertically-tuned RGC inputs may be the major determinant of axis-selectivity for these tectal cells. Indeed, a major advance afforded by the development of RGECO is using two color functional imaging to test hypotheses such as this. Expression of RGECO in single tectal neurons in a background of SyGCaMP3-expressing RGC axons (Nikolaou et al., 2012) will allow for direct correlative studies of pre-and postsynaptic tuning properties. For circuit neuroscience in general, functionally imaging genetically defined pre- and postsynaptic circuit components, separable by color, promises to be a powerful approach to studying neural interactions in the brain.

### Conflict of interest statement

The authors declare that the research was conducted in the absence of any commercial or financial relationships that could be construed as a potential conflict of interest.
